# Further data to address the Alzheimer's coverage with evidence development questions

**DOI:** 10.1002/alz.71724

**Published:** 2026-08-04

**Authors:** Malaz Boustani, Eric G. Klein, Jennifer A. Zimmer, Hong Wang, Staci E. Engle, Adam Phipps, Maureen Japha, Traci Schilling, Ann Hartry, for the Alzheimer's Disease Neuroimaging Initiative

**Affiliations:** ^1^ Indiana University School of Medicine Indianapolis Indiana USA; ^2^ Eli Lilly and Company Indianapolis Indiana USA

**Keywords:** Alzheimer disease, amyloid‐related imaging abnormalities, amyloid‐targeting therapy, anti‐amyloid monoclonal antibody, Centers for Medicare and Medicaid Services, cerebral edema, cognitive and functional decline, coverage with evidence development, dementia, Donanemab, early symptomatic Alzheimer's disease, health outcomes, Medicare, mild cognitive impairment, National Coverage Determination

## Abstract

**INTRODUCTION:**

Medicare patients currently access amyloid‐targeting therapies for Alzheimer's disease through the class‐based National Coverage Determination (NCD) with coverage with evidence development (CED) established in 2022. Evidence addressing the stated CED questions was previously published in a 2024 review; however, the original 2022 NCD remains unchanged.

**METHODS:**

Update the previously published review responding to the CED questions by summarizing recently published donanemab and lecanemab extension data and new safety‐related data.

**RESULTS:**

New evidence addresses each of the three CED questions. Extension data show that the benefit of amyloid‐targeting therapy continues to accrue with no new safety signals observed. Safety analyses identified baseline risk factors for amyloid‐related imaging abnormalities (ARIA). Further, a more gradual donanemab titration regimen decreased risk of ARIA‐edema/effusions.

**DISCUSSION:**

With CED questions addressed, reconsideration of the class‐based NCD is scientifically justified.

**Clinical Trial Registration**: NCT04437511, NCT05738486, NCT03887455

## Highlights

- Amyloid‐targeting therapies show an increasing treatment effect over 3 years.
- Long‐term extension data reinforced the importance of early intervention.
- Analyses of amyloid‐related imaging abnormalities (ARIA) events identified baseline risk factors.
- More gradual titration of donanemab reduced the ARIA with edema/effusion (ARIA‐E) risk.
- New evidence addresses each of the three coverage with evidence development (CED) questions.

## 1 BACKGROUND

Donanemab and lecanemab are amyloid‐targeting therapies (ATTs) approved by the US Food and Drug Administration that slow the progression of Alzheimer's disease (AD).^1^, ^2^ Currently, coverage of these medications is restricted under coverage with evidence development (CED) in the National Coverage Determination (NCD) issued by the Centers for Medicare & Medicaid Services (CMS) in April 2022.^3^ At the suggestion of CMS, Klein et al^4^ published a response in 2024, addressing each of the three questions posed by CMS in the CED with robust evidence for donanemab. Despite the strength of the evidence, CMS has not yet initiated reconsideration of the national coverage policy.

New data available since the Klein et al report are summarized to provide further evidence relevant to CMS's questions. Phase 3 long‐term extension (LTE) and open‐label extension (OLE) data have been published for both donanemab and lecanemab, respectively.^5^, ^6^ Clinical efficacy in these studies was assessed using the Clinical Demetia Rating (CDR) scale, including CDR‐Sum of Boxes (CDR‐SB) and CDR‐Global (CDR‐G). CDR evaluates impairment across cognitive and functional domains relevant and important to individuals living with AD.^7^ CDR‐SB is a continuous measure ranging from 0 to 18, with higher scores indicating greater impairment and is useful for detecting gradual changes over time. In contrast, CDR‐G is a categorical staging measure with values of 0 (normal), 0.5 (mild cognitive impairment/very mild impairment), 1 (mild dementia), 2 (moderate dementia), and 3 (severe dementia). Accordingly, CDR‐SB quantifies the extent of change, whereas CDR‐G helps interpret that change in terms of clinically recognizable disease stage. For example, a change in CDR‐G from 0.5 to 1.0 indicates progression from mild cognitive impairment (MCI) to mild dementia due to AD, reflecting worsening in cognition and daily function.^8^, ^9^ Because progression to the next disease stage on the CDR‐G scale represents a marked decline in cognition and daily function, slowing this progression is considered clinically meaningful.^10^ Further, slowing of disease progression by greater than 25% on the CDR‐SB scale has been recognized as clinically meaningful to clinicians and patients.^11^

In addition to extension efficacy data representing additional follow‐up time, here we also review new studies and evidence related to amyloid‐related imaging abnormalities (ARIA) risk and management.^12^, ^13^, ^14^ 

## 2 UPDATES TO THE CED QUESTION 1 RESPONSE: DOES THE ANTI‐AMYLOID MONOCLONAL ANTIBODY MEANINGFULLY IMPROVE HEALTH OUTCOMES (i.e., SLOW THE DECLINE OF COGNITION AND FUNCTION) FOR PATIENTS IN BROAD COMMUNITY PRACTICE?

Klein et al^4^ previously outlined how phase 3 TRAILBLAZER‐ALZ 2 study participants reflect the Medicare population. Similar comorbidities and co‐medication use were noted between the populations, while ethnic and racial representation were lower in the trial population.^4^ Real‐world evidence now demonstrates that patients treated with donanemab in community practice are largely similar to those in clinical trials. Recent findings indicate that, in community practice, patients enter donanemab treatment at an average age of approximately 77 years and are around 47% male.^15^ These demographics align well with TRAILBLAZER‐ALZ 2.^16^ Additionally, the majority of Mini‐Mental State Examination scores at treatment initiation in the real world and TRAILBLAZER‐ALZ 2 screening fall into categories consistent with AD with MCI or mild dementia. Current observations of community practice indicate a slight reduction in patients homozygous for the apolipoprotein E (*APOE*) *ε*4 allele (5%) compared with TRAILBLAZER‐ALZ 2 (17%).^16^, ^17^ Initial US real‐world evidence of lecanemab shows patients enter treatment at an average age of approximately 75 years and are around 45% male, aligning with the phase 3 Clarity AD trial. However, real‐world data showed fewer lecanemab‐treated patients with an MCI diagnosis (32%) compared with Clarity AD (62%).^18^ Another lecanemab study of real‐world evidence reported similar demographic findings, with the addition of a consistent number of patients homozygous for the *APOE ε*4 allele (around 16% in real‐world data and Clarity AD).^19^, ^20^ Together, these findings show similarities between characteristics of patients treated with ATTs in community practice to date and clinical trial populations, suggesting that clinical trial results are generalizable to community practice. However, in these clinical trials and real‐world populations, ethnic, racial, and socioeconomic status diversity are less than in the Medicare population.^21^

**RESEARCH IN CONTEXT**

1. **Systematic review**: To provide an update on the Klein et al 2024 paper, the authors reviewed new donanemab and lecanemab data that help address the coverage with evidence development questions.
2. **Interpretation**: Additional data from the TRAILBLAZER‐ALZ 2 long‐term extension and the Clarity AD open‐label extension reinforce that the clinical effects of amyloid‐targeting therapy reflect a measurable therapeutic effect, with benefits that continue to increase over time, consistent with disease modification. No new safety signals were identified during the course of the long‐term extension studies, compared to the established safety profile.
3. **Future directions**: The substantial evidence available supports reconsideration of the national coverage policy on amyloid‐targeting therapies, with the potential removal of coverage with evidence development (CED) requirements.

Donanemab and lecanemab treatment slowed cognitive and functional decline in the main, placebo‐controlled portions of TRAILBLAZER‐ALZ 2 and Clarity AD.^16^, ^20^ Expanding on the previous Klein et al report,^4^ clinical meaningfulness of donanemab was reviewed by Atri et al using data from the placebo‐controlled portion of TRAILBLAZER‐ALZ 2. They reported that the risk of a meaningful decline in cognition and function and progression of dementia severity was lower in donanemab‐treated participants. By slowing clinical progression, donanemab treatment may allow patients living with AD to retain a higher degree of independence and continue engaging in activities that matter most to them. The totality of evidence across multiple outcome measures and analytic approaches supports that donanemab's benefits are clinically meaningful for patients and caregivers, not just statistically significant.^22^

**FIGURE 1:**
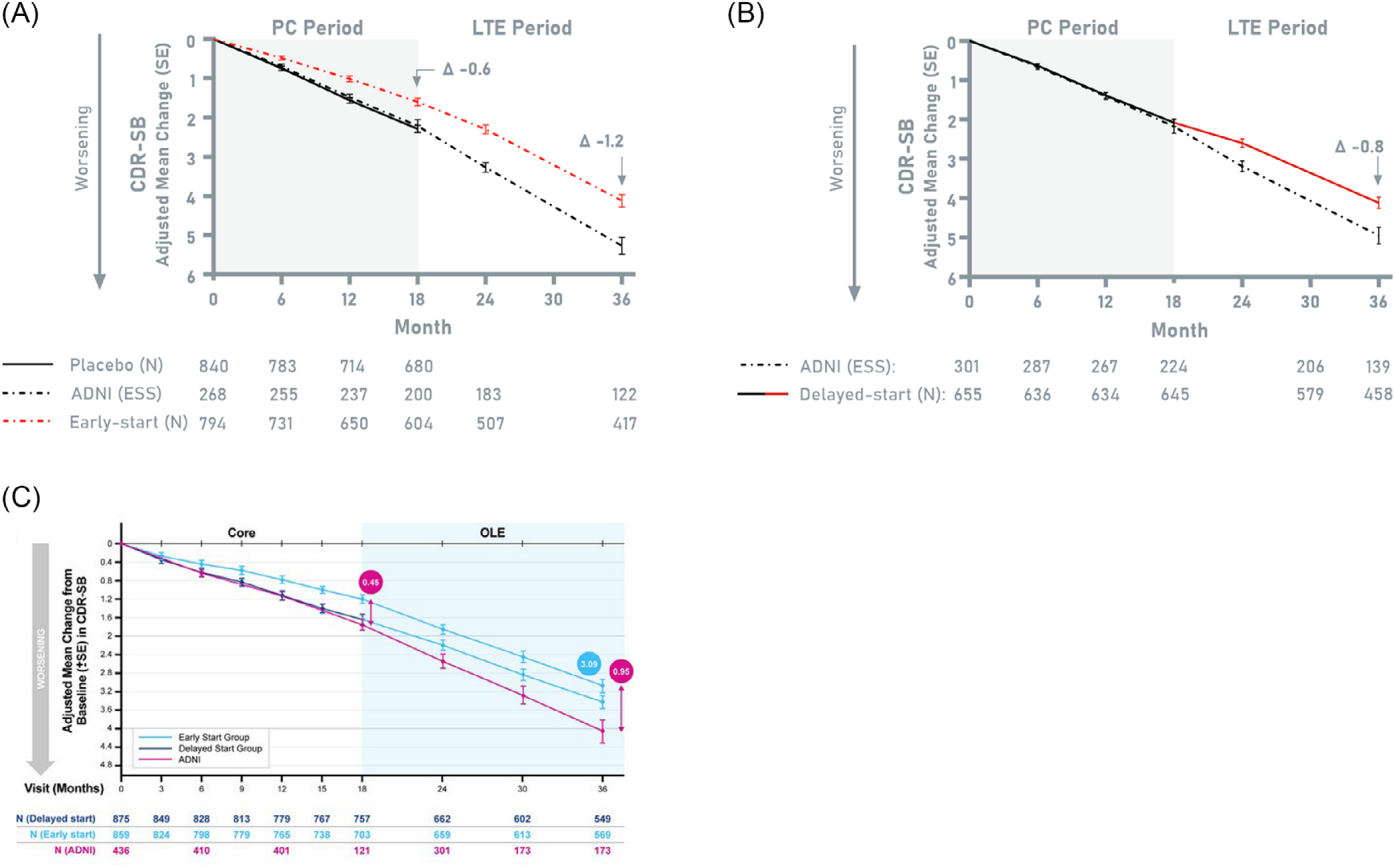
Clinical efficacy by change in CDR‐SB score with donanemab and lecanemab. (A–B) Change from baseline in CDR‐SB score from TRAILBLAZER‐ALZ 2 (placebo‐controlled period and LTE period) comparing early‐start (A) and delayed‐start (B) donanemab‐treated participants to an external propensity‐score weighted ADNI control cohort. Data from Zimmer et al.^6^ (C) Change from baseline in CDR‐SB score from Clarity AD (placebo‐controlled period and OLE period) comparing early and delayed start lecanemab‐treated participants to an external matched ADNI control cohort. Figure from van Dyck et al.^5^ ADNI, Alzheimer's Disease Neuroimaging Initiative; CDR‐SB, Clinical Dementia Rating ‐ Sum of Boxes; ESS, effective sample size; LTE, long‐term extension; N, number of participants; OLE, open‐label extension; PC, placebo‐controlled; SE, standard error.

**FIGURE 2:**
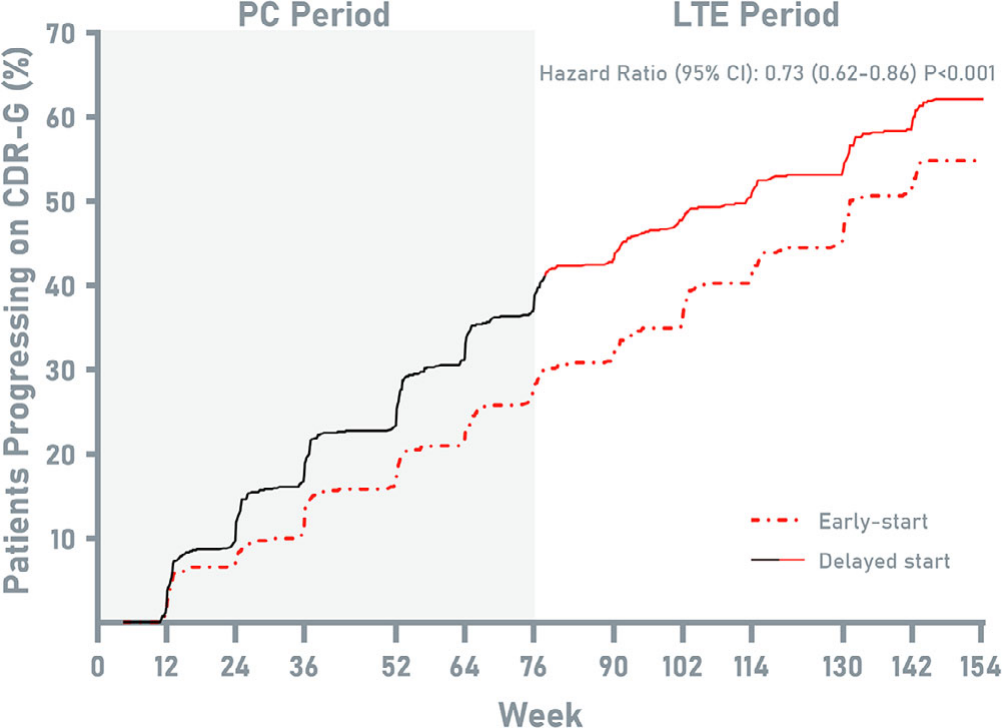
Progression to the next stage of AD. Hazard progression analysis, based on CDR‐G scores, for early‐start versus delayed‐start donanemab participants in TRAILBLAZER‐ALZ 2. Data from Zimmer et al.^6^ AD, Alzheimer's disease; CI, confidence interval; CDR‐G, Clinical Dementia Rating‐Global; LTE, long‐term extension; PC, placebo‐controlled.

Trial extension data are now available for both donanemab and lecanemab, which provide further information on the long‐term efficacy of ATTs.^5^, ^6^ Donanemab data from the TRAILBLAZER‐ALZ 2 LTE demonstrated meaningful slowing of clinical decline with increasing clinical benefits up to 3 years compared with a propensity‐score weighted untreated external Alzheimer's Disease Neuroimaging Initiative (ADNI) control population. These LTE data provide further evidence that donanemab successfully modifies the course of early symptomatic AD. “Early‐start” participants who received donanemab during the main study period and met treatment completion criteria of amyloid clearance started the 78‐week LTE on placebo (71%). “Early‐start” participants that did not meet treatment completion criteria continued donanemab treatment (29%). In contrast, “delayed‐start” LTE participants who received placebo during the main study period were switched to donanemab in the LTE. Among early‐start participants, CDR‐SB scores indicated that cognitive and functional decline was differentiated during the placebo‐controlled period from the weighted ADNI control cohort. CDR‐SB scores continued to diverge over the 78‐week LTE, indicating the disease‐modifying effect of donanemab treatment, with an adjusted mean treatment difference of −1.2 points at 3 years (Figure 1A). Donanemab also slowed clinical decline in the delayed‐start participants, with an adjusted mean treatment difference in the CDR‐SB score of −0.8 points at 3 years (76 weeks after initiating donanemab in the LTE period) compared with the weighted ADNI control cohort (Figure 1B).^6^ In a comparison of early‐ versus delayed‐start groups, using the CDR‐G score (Figure 2), early‐start participants showed a 27% reduced risk of progressing to the next clinical stage of disease versus delayed‐start participants.^6^

Lecanemab Clarity AD OLE data also support that ATTs continue to provide long‐term benefit.^5^ In the 18‐month OLE, Clarity AD participants continued lecanemab treatment (early start participants) or were switched from placebo to lecanemab (delayed start participants). The change in CDR‐SB scores for early start participants continued to diverge from those of the matched external ADNI control cohort throughout the Clarity AD OLE period (adjusted mean treatment difference of −0.95 points between lecanemab and the matched ADNI control cohort at 3 years), indicating treatment benefit (Figure 1C).^5^ Lecanemab‐treated participants demonstrated a reduction in cognitive decline of 1.01 points on the CDR‐SB scale over 3 years of treatment, growing to a reduction of 1.75 points at 4 years, compared to the expected decline in the matched ADNI control cohort, demonstrating a benefit that expands over time.^23^ At 4 years in the OLE, lecanemab‐treated participants had a 34% lower relative risk of progressing to the next disease stage compared with the matched ADNI control cohort.^24^

These efficacy findings from clinical trial participants are informative, showing the clinically meaningful benefits that ATTs provide to patients with comorbidities and co‐medication use similar to those in the Medicare population.

## 3 UPDATES TO THE CED QUESTION #2 RESPONSE: DO BENEFITS AND HARMS, SUCH AS BRAIN HEMORRHAGE AND EDEMA, ASSOCIATED WITH USE OF THE ANTI‐AMYLOID MONOCLONAL ANITBODY, DEPEND ON CHARACTERISTICS OF PATIENTS, TREATING CLINICIANS, AND SETTINGS?

In terms of benefit, data from the placebo‐controlled portion of TRAILBLAZER‐ALZ 2 established that participants characterized as earlier in the course of disease progression derive greater treatment benefit, as previously discussed by Klein et al.^4^ The LTE disease progression analysis builds upon this. By the end of the LTE period, early‐start participants had a 27% lower risk of progressing to the next clinical stage than delayed‐start participants, as assessed in a CDR‐G hazard progression analysis (Figure 2), additionally supporting the importance of an early and accurate diagnosis and timely treatment for appropriate patients.^6^ In both the TRAILBLAZER‐ALZ 2 LTE and Clarity AD OLE, participants in the delayed‐start groups showed a difference in the rate of progression compared to the ADNI control cohort; although this difference was not as robust as that seen in early‐start participants (Figure 1).^5^, ^6^

In terms of harm, ARIA is a known risk of ATTs, occurring in both donanemab‐ and lecanemab‐treated participants, as well as other agents in the class.^12^, ^16^, ^20^ A secondary analysis of TRAILBLAZER‐ALZ and ‐ALZ 2 was conducted to advance the understanding of ARIA risk and mitigation. It included modeling of risk factors to identify baseline characteristics associated with ARIA with edema/effusions (ARIA‐E) and ARIA‐microhemorrhages and hemosiderin deposition (ARIA‐H).^12^ Baseline variables associated with increased ARIA‐E risk include *APOE ε*4 allele number, greater number of microhemorrhages, presence of cortical superficial siderosis, higher amyloid plaque burden, and elevated mean arterial pressure, while antihypertensive medication use was associated with decreased ARIA‐E risk.^12^ The strongest baseline variables predicting ARIA‐H were *APOE* *ε*4 allele number and cortical superficial siderosis.^12^ With US Food and Drug Administration (FDA) approval of donanemab in July 2024, real‐world use has been accumulating and we expect reporting to begin in late 2026.

Real‐world data for lecanemab provide additional information on ARIA occurrence by *APOE* genotype. Interim results from the LEADER real‐world analysis of lecanemab showed ARIA‐E events occurred in approximately 6% of noncarriers, 8% of patients heterozygous for the *APOE ε*4 allele, and 12% of patients homozygous for the *APOE ε*4 allele. ARIA‐E events were mostly asymptomatic and mild‐to‐moderate in severity.^19^ A recent meta‐analysis was conducted to assess ARIA risk in patients taking anticoagulants. Clinical trial data on donanemab and lecanemab and a real‐world study of lecanemab were included. The meta‐analysis did not find an increased ARIA risk in patients treated with both oral anticoagulants and ATTs.^25^

While the updated findings above focus on the characteristics of the patient, the role of the treating clinicians and setting were previously discussed in the initial Klein et al^4^ response. In TRAILBLAZER‐ALZ 2, donanemab was administered in settings resembling real‐world practice, including community‐based clinics and non‐academic medical centers with a range of provider types.^4^ This extends to the LTE, which was conducted in the same settings as the placebo‐controlled portion of TRAILBLAZER‐ALZ 2.^6^

## 4 UPDATES TO THE CED QUESTION #3 RESPONSE: HOW DO THE BENEFITS AND RISKS CHANGE OVER TIME?

TRAILBLAZER‐ALZ 2 LTE and Clarity AD OLE data demonstrate that the benefits of ATTs continue to grow over 3 years (Figure 1).^5^, ^6^ Donanemab data from the TRAILBLAZER‐ALZ 2 LTE show that the benefit of donanemab treatment persists even after treatment course completion based on amyloid clearance criteria. Participants who met treatment completion criteria (amyloid plaque level of < 11 Centiloids (CL) on any single positron emission tomography (PET) scan or < 25 CL on two consecutive PET scans) and were switched to placebo by 52 weeks in the placebo‐controlled portion of the trial had an adjusted treatment difference in the CDR‐SB score of −1.3 versus the weighted ADNI control cohort at the end of the LTE period. This is similar to the treatment difference for the entire early‐start group versus the control cohort.^6^

**FIGURE 3:**
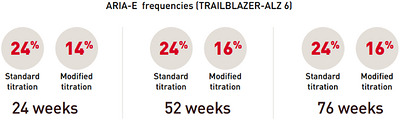
Frequency of ARIA‐E in TRAILBLAZER‐ALZ 6. Comparison of ARIA‐E frequency in TRAILBLAZER‐ALZ 6 for the standard titration regimen (700 mg Q4W for the first three doses followed by 1400 mg Q4W) vs the modified titration regimen (Q4W dosing of 350, 700, 1050, and 1400 mg thereafter) at 24, 52, and 76 weeks.^13^, ^14^. ARIA‐E, amyloid‐related imaging abnormalities with edema/effusion; Q4W, every 4 weeks.

TRAILBLAZER ALZ‐2 and the initially approved prescribing information used a standard titration regimen of donanemab with every 4‐week dosing at 700 mg for the first three doses followed by 1400 mg thereafter (referred to as standard titration).^16^ Subsequently, the TRAILBLAZER‐ALZ 6 trial evaluated ARIA risk in a modified dosing regimen of donanemab with a more gradual titration of every 4‐week dosing at 350, 700, 1050, and then 1400 mg thereafter. The modified titration regimen significantly reduced ARIA‐E risk (24% with standard titration vs. 14% with modified titration by 24 weeks)(Figure 3) while maintaining amyloid reduction.^13^, ^14^ This more gradual titration regimen is now reflected in the approved US prescribing information.^1^ The modified titration regimen did not significantly reduce ARIA‐H or macrohemorrhage compared to the standard regimen; however the study was not designed or powered to detect ARIA‐H outcomes.^14^ To potentially mitigate ARIA risk early in the course of lecanemab treatment, the prescribing information was updated to require an additional, earlier monitoring MRI.^2^, ^26^ Together, these data provide evidence to address how treatment approach can help manage risk over time.

Importantly, findings from the trial extension periods have not only shown that clinical benefit continued to grow, but also that no new safety signals were observed when donanemab or lecanemab were administered over the additional 18 months following the main, placebo‐controlled periods.^5^, ^6^ The safety profile of early‐start participants who continued donanemab treatment in the TRAILBLAZER‐ALZ 2 LTE was comparable to that of placebo‐treated participants in the placebo‐controlled period, with lower ARIA and infusion‐related reaction frequencies occurring during the LTE.^6^, ^16^ Findings from the Clarity AD OLE reported similar ARIA and lower infusion‐related reaction frequencies for delayed‐start lecanemab‐treated participants in the OLE compared to lecanemab‐treated participants in the placebo‐controlled portion of Clarity AD.^20^, ^27^ ARIA‐E occurs most frequently during the first 6 months of ATT use.^12^, ^27^

## 5 DISCUSSION

Since the initial publication by Klein et al^4^ to address the CMS questions, extension data and additional safety data have been reported for donanemab and lecanemab.^5^, ^6^, ^12^, ^13^, ^14^

Phase 3 LTE data highlight the long‐term clinical benefit of donanemab and the value of initiating treatment early. Further, treatment effects beyond the 76‐week placebo‐controlled period, even after treatment completion, suggest that early amyloid clearance (< 24.1 CL) yields sustained disease modification.^6^ Results from the lecanemab OLE corroborate the finding that significantly reducing amyloid burden is associated with clinically meaningful and durable benefits.^5^

The extension studies described provide both efficacy and extended safety monitoring over time. No new safety signals were observed, remaining consistent with the established safety profile.^5^, ^6^ Regarding ARIA risk with ATTs, further analyses identified baseline factors associated with ARIA‐E risk and the potential to improve detection of ARIA with earlier monitoring.^2^, ^12^ Additionally, the frequency and severity of ARIA‐E were assessed with different donanemab dosing regimens in TRAILBLAZER‐ALZ 6. A modified titration regimen with a more gradual titration than the standard protocol significantly reduced ARIA‐E risk and has been incorporated into approved product labeling.^13^, ^14^ Hence, combining patient‐specific risk assessment with the more gradual titration may decrease the risk of ARIA‐E with donanemab use in clinical practice.

## 6 CONCLUSIONS

Both donanemab and lecanemab demonstrate the ability to slow cognitive and functional decline in clinical trial participants, with clinical benefits continuing to increase over time versus a matched comparator cohort in the trial extensions.^5^, ^6^, ^16^, ^20^ The totality of evidence, which now includes long‐term efficacy and safety,^5^, ^6^ extended safety analyses^12^, additional early MRI monitoring with lecanemab,^26^ and a more gradual donanemab titration approach,^13^, ^14^ help address Medicare's CED questions posed in the 2022 NCD. Accordingly, reconsideration of the class‐based NCD is scientifically justified. The emergence of additional real‐world data will provide further evidence regarding these questions.

## CONFLICT OF INTEREST STATEMENT

Eric G. Klein, Jennifer Zimmer, Hong Wang, Staci E. Engle, Adam Phipps, Maureen Japha, Traci Schilling, and Ann Hartry are employees and minor shareholders of Eli Lilly and Company. Malaz Boustani serves as a Chief Scientific Officer and co‐founder of BlueAgilis and the Chief Health Officer of Mozyne Health, Inc. He has equity interest in Blue Agilis, Inc; and Mozyne Health, Inc. He also has equity interest in NeuroX. He sold his equity in Preferred Population Health Management LLC; and MyShift, Inc (previously known as RestUp, LLC). He co‐founded and served as the Chief Health Officer of DigiCare Realized, Inc that was folded in Early 2025 with no financial gain. He serves or served as an advisory board member or consultant for Eli Lilly and Co, Eisai; Inc; Merck & Co Inc; Biogen Inc; and Genentech Inc. These conflicts have been reviewed by Indiana University and has been appropriately managed to maintain objectivity. Author disclosures are available in the Supporting Information.

## CONSENT STATEMENT

All patients gave informed consent for participation in the studies reviewed.

## Supporting information



Supporting Information
